# Supplementary material for: Neuronal pentraxin 2 is required for facilitating excitatory synaptic inputs onto spinal neurons involved in pruriceptive transmission in a model of chronic itch
Source: Nat Commun. 2022 May 2;13:2367. doi: 10.1038/s41467-022-30089-x (PMC9061767; doi:10.1038/s41467-022-30089-x)
Supplement: Supplementary file 1 — Supplementary Information [file 41467_2022_30089_MOESM1_ESM.pdf]

Supplementary Information

Neuronal pentraxin 2 is required for facilitating excitatory synaptic inputs onto spinal neurons involved in pruriceptive transmission in a model of chronic itch

Kensho Kanehisa, Keisuke Koga, Sho Maejima, Yuto Shiraishi, Konatsu Asai, Miho Shiratori-Hayashi, Mei-Fang Xiao, Hirotaka Sakamoto, Paul F. Worley, Makoto Tsuda

This PDF file includes:
Supplementary Figure 1 to 7
Legends for Supplementary Figures

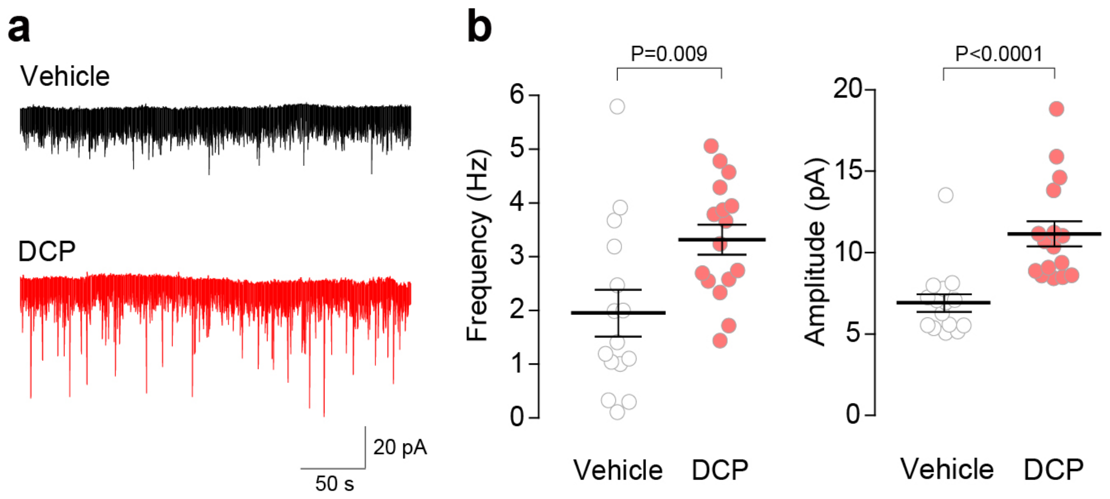

Supplementary Figure 1. Miniature EPSCs in GRPR$^+$ SDH neurons of DCP-treated mice.

a, b Representative traces **(a)** and the average of frequency and amplitude **(b)** of mEPSCs in GRPR$^+$ (mCherry$^+$) SDH neurons of vehicle- and DCP-treated mice in the presence of tetrodotoxin (1 µM) (vehicle, n=15 cells; DCP, n=16 cells). Frequency, unpaired *t* test; amplitude, Mann-Whitney test. Values represent mean ± S.E.M. Source data are provided as a Source Data file.

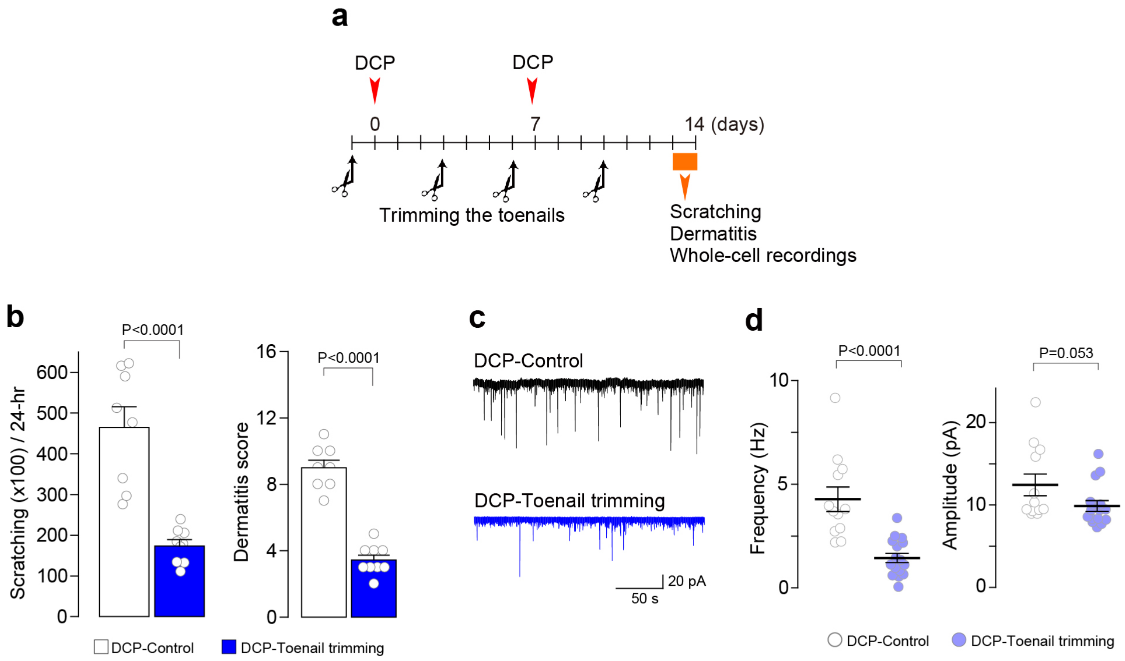

Supplementary Figure 2. Effect of toenail trimming on DCP-induced synaptic facilitation.

a Schematic timeline for trimming the toenails, painting of DCP, measuring scratching behavior and dermatitis score, and whole-cell recordings. **b** Scratching behavior for 24 hr (left) and dermatitis score (right) in DCP-treated mice with or without trimming the toenails (DCP-Control, n=8; DCP-Toenail trimming, n=9; unpaired *t* test). **c, d** Representative traces **(c)** and the average of the frequency and amplitude **(d)** of sEPSCs in GRPR$^+$ (mCherry$^+$) SDH neurons of DCP-treated mice with or without trimming the toenails (DCP-Control, n=12 cells; DCP-Toenail trimming, n=16 cells; frequency, unpaired *t* test; amplitude, Mann-Whitney test). Values represent mean ± S.E.M. Source data are provided as a Source Data file.

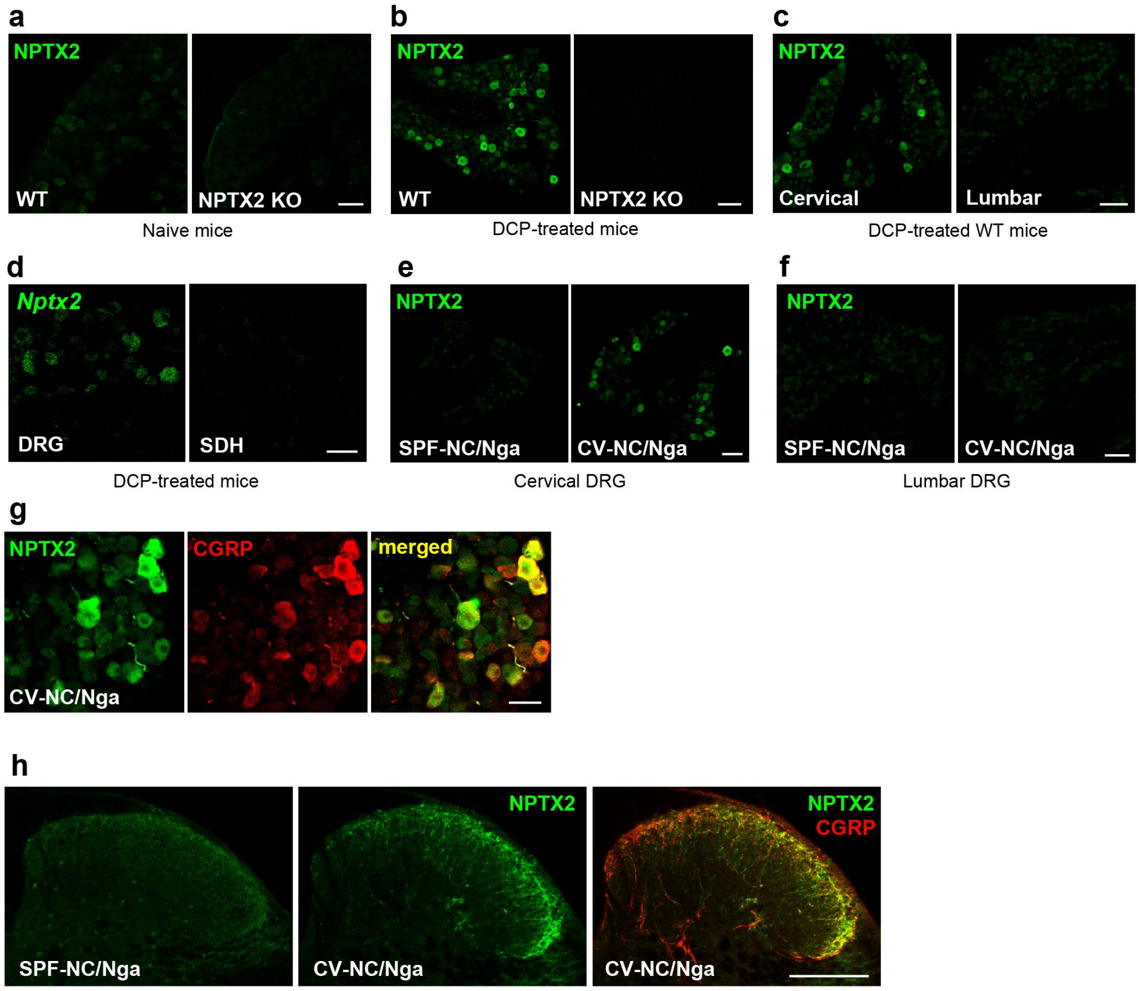

Supplementary Figure 3. NPTX2 immunofluorescence in the cervical DRG and SDH.

a, b NPTX2 immunofluorescence in the cervical DRG of WT and NPTX2 KO mice with **(b)** and without DCP treatment **(a**, naïve**)**. Scale bars, 50 **(a)** and 100 μm **(b)**. **c** NPTX2 immunofluorescence in the cervical or lumbar DRG of DCP-treated mice. Scale bar, 100 μm. **d** RNAscope in situ hybridization for *Nptx2* mRNA (green) in the cervical DRG and SDH of DCP-treated mice. Scale bar, 50 μm. **e, f** NPTX2 immunofluorescence in the cervical **(e)** and lumbar **(f)** DRG in SPF- and CV-NC/Nga mice. Scale bar, 100 μm. **g** Double-immunolabeling of NPTX2 (green) and CGRP (red) in the cervical DRG in CV-NC/Nga mice. Scale bar, 50 μm. **h** NPTX2 immunofluorescence in the cervical SDH of SPF- and CV-NC/Nga. NPTX2 (green) was colocalized with CGRP (red) in the SDH. Scale bar, 200 μm.

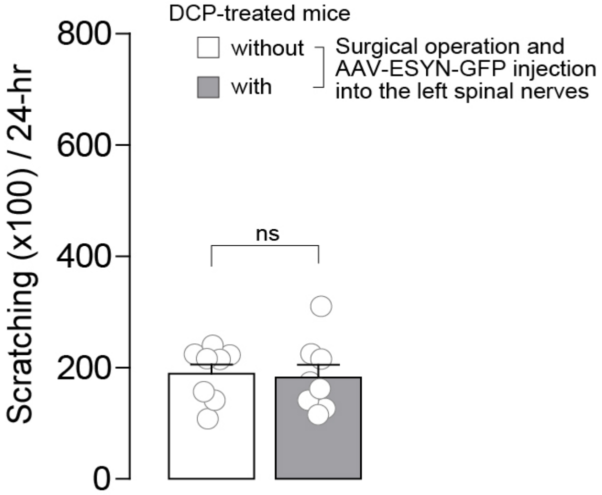

Supplementary Figure 4. Scratching behavior in DCP-treated mice with or without injection of control AAV vector in the left cervical spinal nerves.

Mice were injected with or without the injection of AAV-ESYN-GFP into the left spinal nerves of cervical segments (C3/4) (n=8 mice/group). DCP were topically applied on the skin of the left back, and, 14 days later, scratching behavior by the left hind limb was counted for 24 hr. Values represent mean ± S.E.M. Source data are provided as a Source Data file.

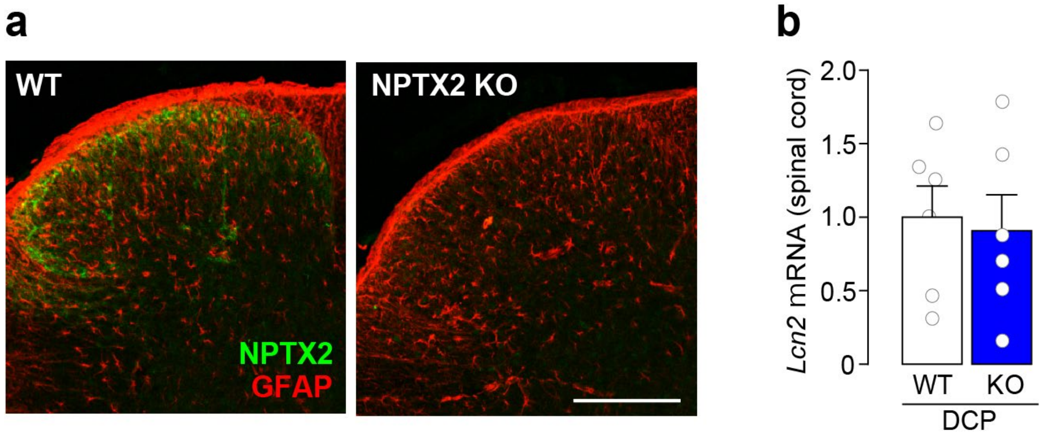

Supplementary Figure 5. No effect of NPTX2 deficiency on DCP-induced activation of astrocytes in the SDH.

a Double-immunolabeling of NPTX2 (green) and GFAP (red) in the cervical SDH of DCP-treated WT and NPTX2 KO mice. Scale bar, 200 µm. **b** *Lcn2* mRNA in the cervical spinal cord in DCP-treated mice (n=6 mice/group). Values represent mean ± S.E.M. Source data are provided as a Source Data file.

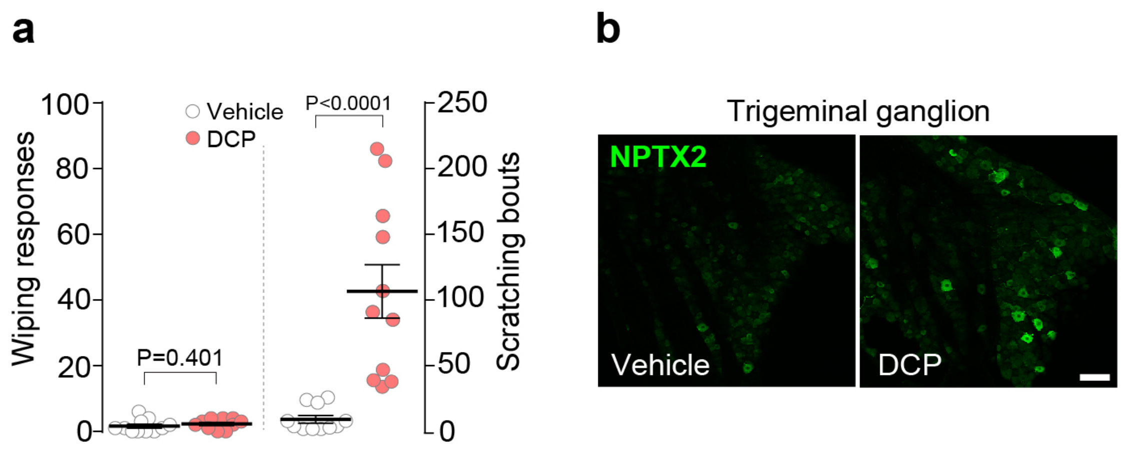

Supplementary Figure 6. Topical application of DCP to the cheek produces scratching, but not wiping behavior.

a DCP or vehicle (acetone) was topically applied to the shaved cheek, and, 14 days later, scratching behavior by hind limbs and wiping behavior by fore limbs directed toward the DCP-treated cheek were video-taped for 30 min and counted (n=11 mice/group). Mann-Whitney test. Values represent mean ± S.E.M. Source data are provided as a Source Data file. **b** Immunofluorescence of NPTX2 in the trigeminal ganglion of vehicle- and DCP-treated mice. Scale bar, 100 μm.

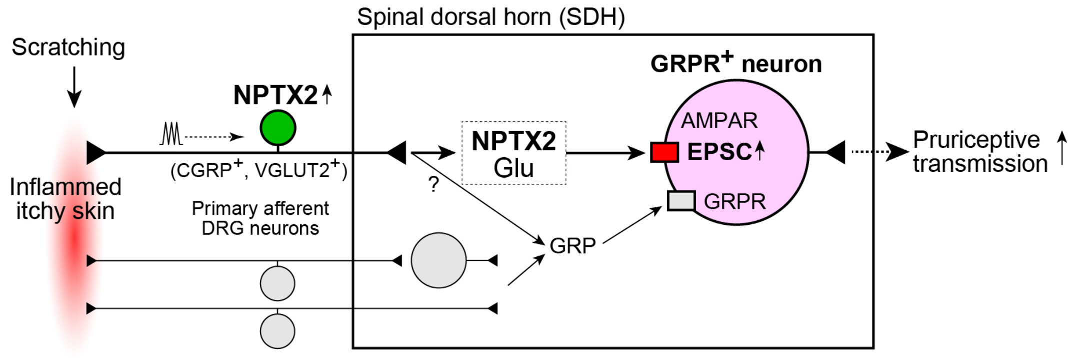

Supplementary Figure 7. A model of the glutamatergic synaptic facilitation in GRPR$^+$ SDH neurons by NPTX2 under chronic itch-like condition.

In mouse models of chronic itch, NPTX2 expression is upregulated in CGRP$^+$ and/or VGLUT2$^+$ DRG neurons that correspond to the inflamed itchy skin. NPTX2 synthesized in the DRG neurons is transported to the central terminals in the SDH and, after release, facilitates EPSCs via an interaction with the AMPARs in GRPR$^+$ SDH neurons. NPTX2-mediated facilitation of glutamatergic excitatory synaptic responses in GRPR$^+$ neurons and GRPR-mediated excitatory responses evoked by GRP derived from primary afferents and/or SDH interneurons may concertedly render GRPR$^+$ SDH neurons more excitable, which is critical for chronic itch-like behavior.
