# Supplementary material for: Neuronal pentraxin 2 is required for facilitating excitatory synaptic inputs onto spinal neurons involved in pruriceptive transmission in a model of chronic itch
Source: Nat Commun. 2022 May 2;13:2367. doi: 10.1038/s41467-022-30089-x (PMC9061767; doi:10.1038/s41467-022-30089-x)
Supplement: Supplementary file 3 — Reporting Summary [file 41467_2022_30089_MOESM3_ESM.pdf]

nature research

Corresponding author(s): Makoto Tsuda

Last updated by author(s): Mar 14, 2022

Reporting Summary

Nature Research wishes to improve the reproducibility of the work that we publish. This form provides structure for consistency and transparency in reporting. For further information on Nature Research policies, see our Editorial Policies and the Editorial Policy Checklist.

Statistics

For all statistical analyses, confirm that the following items are present in the figure legend, table legend, main text, or Methods section.

n/a	Confirmed	
☐	☒	The exact sample size (*n*) for each experimental group/condition, given as a discrete number and unit of measurement
☐	☒	A statement on whether measurements were taken from distinct samples or whether the same sample was measured repeatedly
☐	☒	The statistical test(s) used AND whether they are one- or two-sided<br>*Only common tests should be described solely by name; describe more complex techniques in the Methods section.*
☒	☐	A description of all covariates tested
☐	☒	A description of any assumptions or corrections, such as tests of normality and adjustment for multiple comparisons
☐	☒	A full description of the statistical parameters including central tendency (e.g. means) or other basic estimates (e.g. regression coefficient) AND variation (e.g. standard deviation) or associated estimates of uncertainty (e.g. confidence intervals)
☐	☒	For null hypothesis testing, the test statistic (e.g. *F*, *t*, *r*) with confidence intervals, effect sizes, degrees of freedom and *P* value noted<br>*Give P values as exact values whenever suitable.*
☒	☐	For Bayesian analysis, information on the choice of priors and Markov chain Monte Carlo settings
☒	☐	For hierarchical and complex designs, identification of the appropriate level for tests and full reporting of outcomes
☒	☐	Estimates of effect sizes (e.g. Cohen's *d*, Pearson's *r*), indicating how they were calculated

Our web collection on statistics for biologists contains articles on many of the points above.

Software and code

Policy information about availability of computer code

Data collection	A computer-controlled amplifier (Axopatch 700B, Molecular Devices, CA, USA) for recording of synaptic currents.<br>An analog-to-digital converter (Digidata 1550, Molecular Devices) for digitizing and a data acquisition program (pCLAMP 10.4 acquisition software, Molecular Devices) for storation to a personal computer.<br>Zen 2012 (version 8.1.9.484, Carl Zeiss, Oberkochen, Germany) for acquisition of the fluorescent image.<br>MicroAct software (version 2.14, NEUROSCIENCE) for recording and amplifying of magnetic-induced electric currents in the coil .<br>LightCycler 96 (version 101.01.0050, Roche molecular Systems, Inc) for acquisition of real-time PCR-based quantitative data .
Data analysis	Software package (Clampfit version 10.4, Molecular Devices) and Mini Analysis Program (version 6.0.7, Synaptosoft) for the analysis of frequency and amplitude of EPSCs.<br>Graph Pad Prism 7 (Graph Pad, CA, USA) for all statistical analyses.<br>Zen 2012 (version 8.1.9.484, Carl Zeiss, Oberkochen, Germany) for measurements of fluorescent intensity and quantification of targeting cells.<br>MicroAct software (version 2.14, NEUROSCIENCE) for the analysis of scratching behavior.<br>LightCycler 96 Software (Version 1.1.0.1320, Roche Diagnostics International Ltd) for the analysis of mRNA expression.

For manuscripts utilizing custom algorithms or software that are central to the research but not yet described in published literature, software must be made available to editors and reviewers. We strongly encourage code deposition in a community repository (e.g. GitHub). See the Nature Research guidelines for submitting code & software for further information.

Data

Policy information about availability of data

All manuscripts must include a data availability statement. This statement should provide the following information, where applicable:

- Accession codes, unique identifiers, or web links for publicly available datasets
- A list of figures that have associated raw data
- A description of any restrictions on data availability

Data underlying the findings of the study are available from the corresponding author upon request. Source data is provided with this paper.

Field-specific reporting

Please select the one below that is the best fit for your research. If you are not sure, read the appropriate sections before making your selection.

[x] Life sciences [ ] Behavioural & social sciences [ ] Ecological, evolutionary & environmental sciences

For a reference copy of the document with all sections, see nature.com/documents/nr-reporting-summary-flat.pdf

Life sciences study design

All studies must disclose on these points even when the disclosure is negative.

Sample size	No statistical methods were used to predetermine sample sizes, but the sample size was determined based on our experience with the experimental models and our reported paper (Shiratori-Hayashi et al., Nat Med, 2015; Kohro et al., Sci Rep, 2015; Koga et al., J Allergy Clin Immunol, 2020).
Data exclusions	No data exclusions were applied.
Replication	All experiments has been successfully repeated at least two times.
Randomization	Mice were randomly allocated to groups in all experiments.
Blinding	The investigations were not blinded to allocation during experiments and outcome assessment, because they needed to record the identifier of the sample or animal.

Reporting for specific materials, systems and methods

We require information from authors about some types of materials, experimental systems and methods used in many studies. Here, indicate whether each material, system or method listed is relevant to your study. If you are not sure if a list item applies to your research, read the appropriate section before selecting a response.

Materials & experimental systems

n/a	Involved in the study	
[ ]	[x]	Antibodies
[ ]	[x]	Eukaryotic cell lines
[x]	[ ]	Palaeontology and archaeology
[ ]	[x]	Animals and other organisms
[x]	[ ]	Human research participants
[x]	[ ]	Clinical data
[x]	[ ]	Dual use research of concern

Methods

n/a	Involved in the study	
[x]	[ ]	ChIP-seq
[x]	[ ]	Flow cytometry
[x]	[ ]	MRI-based neuroimaging

Antibodies

Antibodies used	Antibody information about supplier name and catalog number are listed in current paper. Other information are listed below. mouse anti-NeuN (1:2000; ab104224, Abcam, UK, Lot#: GR3255973-4), rabbit anti-PAX2 (1:1000; 71-6000, Invitrogen, Lot#: 1440099A), goat anti-CGRP (1:2000; ab36001, Abcam, Lot#: GR253001-13), rat anti-TRKA (1:500; AF1056, R&D Systems, Lot#: VFA011712A), guinea pig anti-TRPV1 (1:2000; GP14100, NEUROMICS, Lot#: 402241), and biotinylated IB4 conjugates (1:2000; I21414, Invitrogen, Lot#: 1853650), chicken NF200 (1:1000; CH23015, NEUROMICS, Lot#: 403406), guinea pig anti-VGLUT2 (guinea pig anti-VGLUT2 (1:500; Af810, Frontier Institute), Lot#: MSFR106280), rat anti-GFAP (1:2000; 13-0300, Invitrogen, Lot#: QG216413) and rabbit anti-NPTX2 that is not commercially available and is provided by Prof. Paul Worley at Johns Hopkins University School of Medicine.
Validation	The NeuN, PAX2, TRKA, biotinylated IB4 conjugates, GFAP antibody are widely used, and were validated in our previous studies: Masuda et al., Nat Commun 7: 12529 (2016).

Koga et al., Sci Rep 7: 4739 (2017).
Tashima et al., eNeuro 5: ENEURO.0450-17.2018 (2018).
Shiratori-Hayashi et al., Nat Med 21: 927-31 (2015).
The CGRP antibody:Wang et al., Nat Commun 9: 1529 (2018), Usoskin D et al., Nat Neurosci 18: 145-53 (2015).
The TRPV1 antibody: Barry DM et al., Nat Commun 11: 1397 (2020).
The NF200 antibody: Yiting Liu et al., Acta Neuropathol Commun 5: 25 (2017).
The VGLUT2 antibody: Liu Y et al., J Neurosci 35: 9336-55 (2015).
The NPTX2 antibody: Miskimon M et al., J Neuroimmunol 274: 86-95 (2014).

Eukaryotic cell lines

Policy information about cell lines

Cell line source(s)	HEK293T was obtained from ATCC.
Authentication	HEK293T was authenticated by ATCC, and no further authentication was performed for this cell line in this study.
Mycoplasma contamination	HEK293T was negative to mycoplasm contamination.
Commonly misidentified lines (See ICLAC register)	No commonly misidentified cell lines were used in the study.

Animals and other organisms

Policy information about studies involving animals; ARRIVE guidelines recommended for reporting animal research

Laboratory animals	As stated in the Method section, male C57BL/6 mice (Clea, Japan) and male SPF and CV-NC/Nga mice (SLC Japan) were used. NPTX2 KO mice were provided by Prof. Paul Worley. All mice used were 8-15 weeks of age at the start of each experiment and were housed at temperature and humidity ranges of 21 to 23°C and 40 to 60%, respectively with a 12-h light-dark cycle. All animals were fed food and water ad libitum. All animals were housed in standard polycarbonate cages in groups of same-sex littermates.
Wild animals	We did not use wild animals.
Field-collected samples	We did not use field-collected samples.
Ethics oversight	All animal experiments were conducted according to relevant national and international guidelines contained in the 'Act on Welfare and Management of Animals' (Ministry of Environment of Japan) and 'Regulation of Laboratory Animals' (Kyushu University) and under the protocols approved by the Institutional Animal Care and Use committee review panels at Kyushu University.

Note that full information on the approval of the study protocol must also be provided in the manuscript.
